# Interaction Mechanisms of KRAS G12C Inhibitors (Sotorasib and Adagrasib) with Human Serum Albumin: Insights from Spectroscopic and Molecular Docking Studies

**DOI:** 10.3390/molecules30163436

**Published:** 2025-08-20

**Authors:** Junsong Qin, Zhepeng Chen, Chuangyan Wang, Lin Mai, Xian Wang, Junfeng Li, Hui Liu, Yun Song

**Affiliations:** 1 Engineering Research Center of Tropical Medicine Innovation and Transformation of Ministry of Education, Hainan Provincial Key Laboratory of Research and Development on Tropical Herbs, School of Pharmacy, Hainan Medical University, Haikou 571199, China; 2 Key Laboratory of Tropical Translational Medicine of Ministry of Education, Key Laboratory of Brain Science Research Transformation in Tropical Environment of Hainan Province, School of Basic Medicine and Life Sciences, Hainan Medical University, Haikou 571199, China

**Keywords:** KRAS G12C, Sotorasib, Adagrasib, human serum albumin, fluorescence titration, molecular docking

## Abstract

This study systematically explored the interaction mechanisms between two KRAS G12C inhibitors (Sotorasib and Adagrasib) and human serum albumin (HSA) via UV-vis spectroscopy, fluorescence spectroscopy, three-dimensional fluorescence spectroscopy, and molecular docking methods. The experimental findings demonstrated that both drugs caused static quenching of HSA fluorescence, with binding constants of 13.64 × 10^3^ M^−1^ (Sotorasib) and 63.67 × 10^3^ M^−1^ (Adagrasib), demonstrating significant selectivity differences in their binding affinities. UV spectral analysis demonstrated distinct microenvironmental perturbations: Sotorasib and Adagrasib induced a shift (∆λ = 7 nm and ∆λ = 8 nm, respectively) at 211 nm, consistent with altered polarity in HSA’s binding pockets. Fluorescence spectroscopy confirmed a 1:1 binding stoichiometry, with Stern-Volmer analysis validating static quenching as the dominant mechanism. Three-dimensional fluorescence spectra further highlighted Adagrasib’s stronger conformational impact, reducing tyrosine and tryptophan residue fluorescence intensities by 16% (Peak 1) and 10% (Peak 2), respectively, compared to Sotorasib. Molecular docking revealed divergent binding modes: Sotorasib occupied Sudlow Site I via three hydrogen bonds and hydrophobic interactions (∆G = −24.60 kJ·mol^−1^), whereas Adagrasib bound through one hydrogen bond and hydrophobic forces (∆G = −30.92 kJ·mol^−1^), with stability differences attributed to structural characteristics. This study uses multispectral technology and molecular docking to reveal the binding mechanism of Sotorasib and Adagrasib with HSA, providing a theoretical basis for designing highly targeted albumin nanocarriers. The strong binding properties of Adagrasib and HSA may reduce the toxicity of free drugs, providing direction for the development of long-acting formulations.

## 1. Introduction

**Figure 1:**
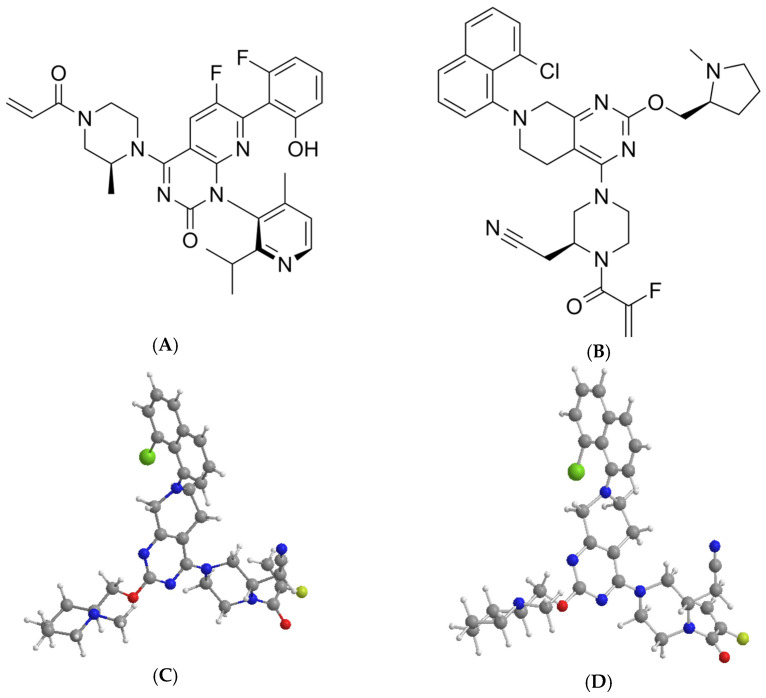
Chemical structure of drugs, Sotorasib (**A**,**C**) and Adagrasib (**B**,**D**).

As a member of the RAS gene family, the Kirsten rat sarcoma viral oncogene homolog (KRAS) encodes a small GTPase protein that functions as a molecular switch in cellular signaling pathways. Notably, it represents the most frequently mutated oncogene in human cancers, with KRAS G12C mutations accounting for approximately 13% of lung adenocarcinoma cases. Sotorasib and Adagrasib (Figure 1) have emerged as potent and selective inhibitors targeting the KRAS G12C mutant protein [1], demonstrating remarkable clinical efficacy through covalent modification of the mutated cysteine residue. Their distinct molecular architectures, as illustrated in Figure 1, underlie differential binding kinetics and therapeutic profiles observed in preclinical studies.

As second generation KRAS G12C inhibitors, Sotorasib and Adagrasib exhibit distinct pharmacological profiles. Sotorasib, an orally bioavailable first-in-class agent [2], achieves selective inhibition through irreversible covalent binding to cysteine 12 in GDP-bound KRAS G12C. This interaction traps the oncoprotein in an inactive state, suppressing KRAS-dependent signaling pathways critical for cellular proliferation (IC50 0.004–0.032 μm in KRASG12C models vs. >7.5 μm in wild-type cells) [3]. Adagrasib effectively inhibits KRAS G12C by covalently binding to the mutant cysteine within KRAS G12C, thereby locking it in an inactive state and preventing downstream signaling, without affecting the wild-type KRAS protein [4]. Despite their clinical approval for non small cell lung cancer (13% KRASG12C prevalence), therapeutic utility remains constrained by acquired resistance, toxicity profiles (Adagrasib associated gastrointestinal/hepatic effects) [5,6], and biomarker stratification challenges.

Nanocarrier encapsulation presents a strategic approach to mitigate toxicity and enhance pharmacokinetics. While existing platforms (liposomes, polymeric micelles, nanoparticles) face limitations in stability and payload capacity, serum albumin (SA) emerges as an optimal biomaterial. Accounting for 55–65% of plasma proteins, SA exhibits exceptional biocompatibility, prolonged circulation, and inherent tumor targeting via SPARC-mediated uptake [7,8,9]. The existing liposome carriers have the problem of insufficient stability, while albumin can improve drug enrichment efficiency through the SPARC-mediated tumor uptake mechanism. Albumin-based formulations (nanoparticles, drug conjugates) demonstrate particular promise in oncology by improving solubility and tumor accumulation [10]. At present, there is a lack of thermodynamic and structural analysis of Sotorasib/Adagrasib binding to human serum albumin (HSA), which hinders the optimization of albumin nanoparticles. Elucidating Sotorasib/Adagrasib–HSA interactions proves critical for nanocarrier optimization. Our multimodal investigation integrates UV-Vis spectroscopy, fluorescence analysis [11], molecular docking [12], and Job plot methodology to characterize the following: 1. binding thermodynamics (fluorescence-derived association constants); 2. structural binding motifs (docking-identified hydrogen bonds/hydrophobic interfaces); 3. stoichiometric ratios (Job plot validation of 1:1 complexes). This mechanistic profiling enables rational design of albumin nanovehicles with enhanced drug loading, controlled release kinetics, and improved tumor specificity. The established structure–activity relationships provide a computational framework for developing next-generation KRAS inhibitors with optimized albumin-binding characteristics and therapeutic indices [13,14].

## 2. Results and Discussion

### 2.1. UV-Vis Absorption Spectroscopic Analysis

UV-Visible spectroscopy served as a critical tool for detecting structural perturbations in HSA and monitoring drug–protein complex formation [15]. The absorption spectrum of HSA exhibits two distinct electronic transitions: a strong peptide backbone absorption at 210 nm and a weaker broad band at 280 nm attributed to aromatic residues. Drug binding induces conformational reorganization of HSA, manifesting as hypochromic/hyperchromic shifts and wavelength displacement (bathochromic or hypsochromic) at 210 nm. Notably, a bathochromic shift (red shift) reflects increased polarity around tryptophan/tyrosine microenvironments, whereas a hypsochromic shift (blue shift) indicates enhanced hydrophobicity or reduced solvent accessibility of these aromatic systems [16]. These spectral signatures provide real-time insights into drug-induced tertiary structural modifications of the albumin carrier.

**Figure 2:**
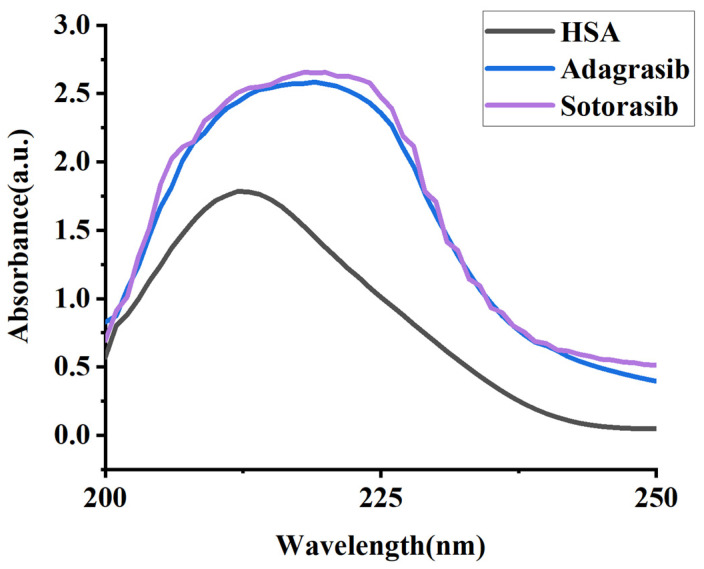
UV spectra of HSA (1.0 × 10^−6^ mol/L), and HSA (1.0 × 10^−6^ mol/L) in the presence of Sotorasib and Adagrasib in aqueous phosphate-buffer solution at 298.15 K. The concentration of Sotorasib and Adagrasib is 4.8 × 10^−6^ mol/L.

UV-Vis spectral analysis of HSA (1.0 × 10^−6^ M) demonstrated concentration-dependent shifts upon interaction with Sotorasib and Adagrasib (Figure 2). Quantitative analysis (Table 1) revealed that Sotorasib and Adagrasib induced a shift (∆λ = 7 nm and ∆λ = 8 nm, respectively) at 211 nm., indicating distinct drug-induced electronic perturbations in the HSA binding pockets. These spectral modifications resulted from drug–HSA complex formation [17,18], which altered the electronic transitions of aromatic residues through microenvironmental reorganization.

The observed bathochromic shifts indicate an increase in polarity around aromatic amino acid residues (such as tryptophan, tyrosine, and phenylalanine). The different degrees of bathochromic shifts are likely associated with the specific binding affinities and interaction modes of each drug with HSA. The significant bathochromic shift (∆λ = 8 nm) induced by Adagrasib suggests more stable or extensive interactions with HSA, whereas the slight bathochromic shift (∆λ = 7 nm) of Sotorasib indicates minimal microenvironmental changes in HSA, reflecting a slight increase in polarity.

### 2.2. Fluorescence Spectroscopy Analysis

Fluorescence spectroscopy was employed to characterize drug-HSA binding dynamics through intrinsic fluorescence monitoring of tryptophan/tyrosine residues [19,20]. To overcome inherent experimental artifacts, particularly inner filter effects (IFEs) caused by high drug concentrations (up to 16.5 × HSA molar ratio), we implemented rigorous methodological controls: (1) conducting titrations in phosphate buffer (pH 7.40) mimicking physiological conditions; (2) optimizing optical pathlength through preliminary calibration studies; (3) maintaining HSA concentration at 2.0 × 10^−6^ M to ensure linear detector response [21]; (4) conducting two rounds of repeated experiments to ensure the achievement of optimal results.

**Figure 3:**
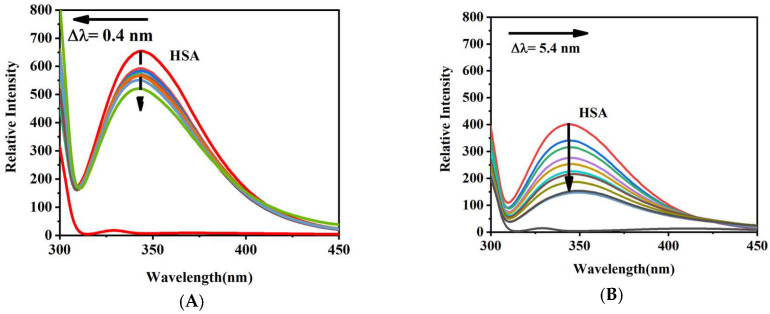
Changes in the fluorescence spectra of HSA (2 × 10^−6^ M) upon the addition of drugs (0–33 × 10^−6^ Μ) in aqueous phosphate-buffer solution at 298.15 K. λex = 295 nm. (**A**) Sotorasib; (**B**) Adagrasib.

As shown in Figure 3, progressive fluorescence quenching was observed with increasing drug concentrations, confirming host–guest complex formation within HSA’s binding cavities [22]. The systematic intensity reduction followed modified Stern-Volmer kinetics, suggesting that static quenching dominated the interaction, a finding corroborated by lifetime measurements in our previous studies [23].

The fluorescence spectral profiles unveiled drug-specific conformational remodeling of HSA through distinct wavelength shifts (Figure 3). As illustrated in Figure 3, Sotorasib induces a subtle 0.4 nm hypsochromic shift (λmax 338.0→337.6 nm), indicating increased hydrophobicity surrounding tryptophan residues within HSA’s binding pocket. Notably, Adagrasib elicits a more pronounced 5.4 nm bathochromic shift (λmax 338.0→343.4 nm), indicating enhanced polarity in the microenvironment of adjacent amino acids. This observation aligns with our molecular docking results demonstrating stabilized complex formation through deeper penetration of Adagrasib’s flexible acrylamide linker into the hydrophobic subdomain of HSA Site I.

### 2.3. Stoichiometry of Drug-HSA Complexes

**Figure 4:**
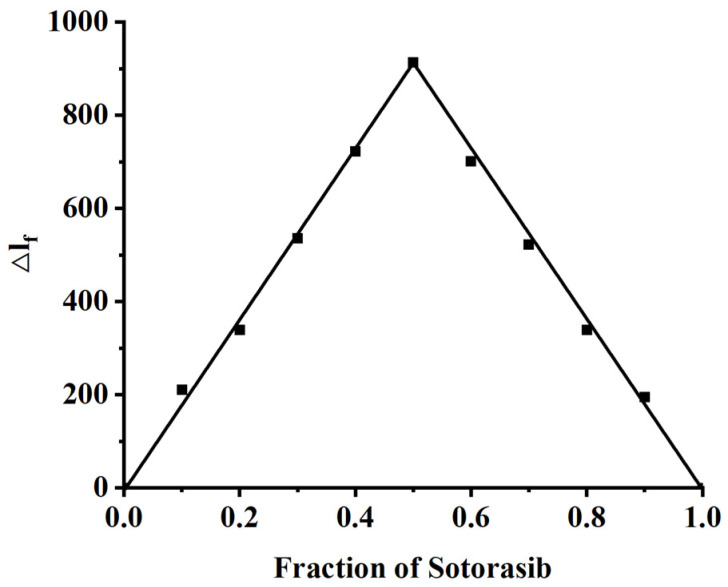
Job’s plot for the complexation of HSA with Sotorasib at 298.15 K in aqueous phosphate-buffer solution ([HSA] + [Sotorasib] = 2.0 × 10^−6^ M), produced with data taken from fluorescence spectra (λex = 295 nm).

The Job plot method (also known as the method of continuous variation) is an analytical technique employed to determine the stoichiometric ratio of complexes formed through molecular interactions [24]. In this experimental design, the total molar concentration (T = [HSA] + [Sotorasib]) was maintained constant at 2.0 × 10^−6^ M, while systematically varying the molar ratio of the two components. Fluorescence spectral analysis conducted under these conditions yielded significant findings. As illustrated in Figure 4, the maximum inflection point of Job’s plot occurred at a mole fraction of 0.5, unambiguously indicating the formation of a 1:1 stoichiometric complex. This quantitative determination reveals that Sotorasib occupies a single binding site on HSA, with a binding stoichiometry (*n*) of 1. The established 1:1 binding ratio provides critical insights into the interaction mechanism between Sotorasib and HSA, demonstrating that the plasma protein exhibits equimolar binding capacity for the therapeutic compound. This stoichiometric relationship has important implications for understanding drug transport and distribution characteristics in physiological systems.

### 2.4. Quenching Mechanism

Fluorescence quenching mechanisms during molecular interactions between small molecules (ligands) and proteins can be classified into three distinct categories: static quenching, dynamic quenching, and combined quenching [25]. Static quenching occurs through ground-state complex formation between fluorophores and quenchers, while dynamic quenching arises from collisional encounters during the excited-state lifetime. Combined quenching incorporates elements of both mechanisms, involving both collisional interactions and stabilized complex formation. These fundamental processes are quantitatively described by the Stern–Volmer equation [26]:

$$F_{0}/F=1+K_{q}\tau_{0}\left[Q\right]=1+K_{\mathrm{sv}}\left[Q\right]$$

where F_0_ and F represent fluorescence intensities in the absence and presence of a quencher, respectively; K_SV_ denotes the Stern-Volmer quenching constant; [Q] is the quencher concentration; K_q_ represents the bimolecular quenching constant; and τ_0_ indicates the fluorescence lifetime (6.3 × 10^−9^ s) [27], a characteristic parameter of the fluorophore.

**Figure 5:**
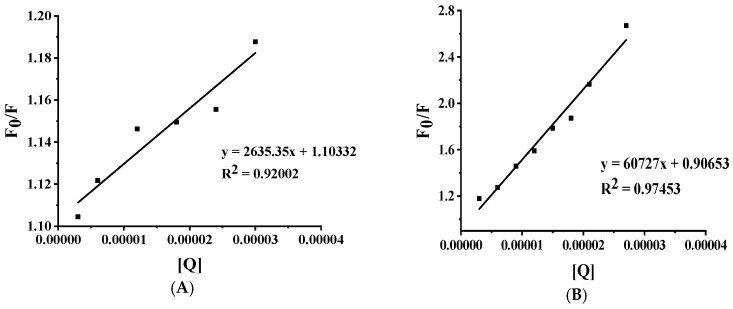
Stern-Volmer plot depicting the interaction between drugs and HSA. (**A**) Sotorasib; (**B**) Adagrasib.

Systematic investigation of HSA fluorescence intensity modulation by therapeutic compounds at varying concentrations (0−33 μm) was conducted through Stern-Volmer analysis. Figure 5 presents the characteristic linear correlation between F_0_/F and [Q], where the X-axis represents quencher concentration (Q) and the Y-axis displays the fluorescence intensity ratio. Experimental data points demonstrate strong agreement with linear regression (R^2^ > 0.90 in all cases, Table 1), confirming predominant static quenching behavior within the tested concentration range. The linear fit parameters, detailed in Table 2, demonstrate excellent goodness of fit, supporting the validity of the proposed quenching mechanism.

The quenching constants (K_SV_), derived from the slopes of linear regression analyses based on the Stern-Volmer equation, were determined to be 2.64 × 10^3^ M^−1^ for Sotorasib and 60.73 × 10^3^ M^−1^ for Adagrasib. These K_SV_ values quantitatively reflect the fluorescence quenching efficiencies resulting from the binding interactions between the respective drugs and HSA.

The experimental data revealed that Sotorasib exhibits a relatively low Stern-Volmer quenching constant (K_SV_), indicative of weaker binding affinity to HSA. This reduced affinity may result in a higher proportion of free drug, potentially leading to accelerated metabolic clearance and a shorter therapeutic duration. Although the bimolecular quenching rate constant (K_q_ = 4.18 × 10^11^ M^−1^ s^−1^) suggests static quenching as the predominant mechanism, the deviation of the intercept (1.10 ± 0.01) from the theoretical value of 1 implies a potential mixed quenching mechanism involving both static and dynamic processes. Furthermore, the slightly lower goodness of fit (R^2^ = 0.92) for the Stern-Volmer equation indicates minor deviations from linearity, which may be attributed to microenvironmental heterogeneity or non-uniformity in complex formation.

In contrast, Adagrasib demonstrated significantly stronger binding characteristics: its K_SV_ value (approximately 22.3-fold higher than that of Sotorasib) and bimolecular quenching rate constant (K_q_ =96.40 × 10^11^ M^−1^ s^−1^) substantially exceeded the dynamic quenching threshold (~1.0 × 10^10^ L·mol^−1^·s^−1^), unequivocally supporting a purely static quenching mechanism. The high linearity of the experimental data (R^2^ = 0.97) and the close agreement of the intercept (0.91 ± 0.06) with the theoretical value of 1 further validated the homogeneity and stability of the binding process. This strong binding affinity is likely to result in an extended plasma half-life and reduced free drug concentration. However, it necessitates caution regarding potential competitive displacement risks with other high-affinity HSA-bound drugs.

The distinct binding profiles of these compounds suggest important pharmacological implications: Adagrasib’s tight binding may offer superior pharmacokinetic controllability, while Sotorasib’s weaker interaction could facilitate enhanced tissue penetration in certain therapeutic contexts. Consequently, the selection of an optimal therapeutic agent should be guided by a careful balance between sustained systemic exposure and target tissue accessibility, tailored to specific clinical objectives.

### 2.5. Binding Constants Between HSA and Drugs

This study quantitatively assessed the interaction strength between HSA and target drugs through fluorescence titration measurements. The binding constant (K_b_), which characterizes the affinity of this molecular interaction, was determined using the following fundamental equation:

$${\log(F}_{0}{\;-\;F)/F\;=\;\mathrm{logK}}_{b}\;+\;\mathrm{nlog}[Q]$$

**Figure 6:**
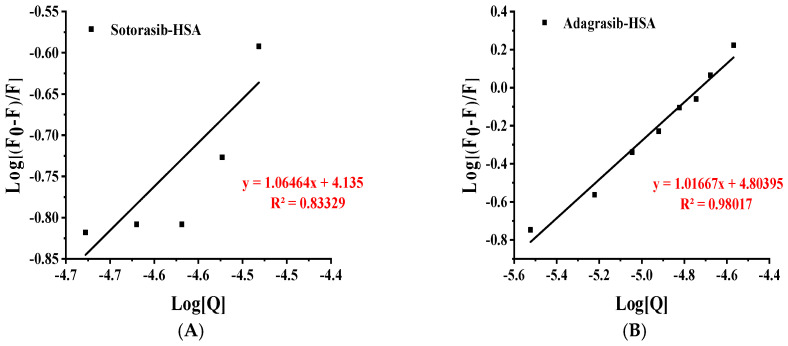
Double logarithmic plot of log(F_0_ − F)/F against log[Q] for the fluorescence quenching data shown in Figure 3, (**A**) Sotorasib; (**B**) Adagrasib.

In this context, K_b_ denotes the binding constant, *n* represents the number of binding sites per HSA molecule, F_0_ and F correspond to the fluorescence intensities without and with quencher addition, respectively, and [Q] signifies the quencher concentration. Figure 6 illustrates a Stern-Volmer-type plot of log[Q] versus log[(F_0_ − F)/F]. Linear regression analysis of the data yielded a straight line, where the slope directly corresponds to *n* (the number of binding sites) and the intercept represents logK_b_.

The free energy change (∆G) for the interaction between the drug and HSA can be calculated using the following equation:

$${\Delta G\;=\;-\mathrm{RTlnK}}_{b}$$

Within the formula, R denotes the gas constant (8.314 J·mol^−1^·K^−1^), T signifies the absolute temperature (298 K, corresponding to 25 °C), and Kb indicates the computed binding constant. A binding site quantity of approximately 1 (*n* ≈ 1) indicates that each HSA molecule interacts with roughly one drug molecule on average, consistent with the 1:1 stoichiometry determined via Job’s method. Fitted data and calculated parameters (K_b_, ∆G) are presented in Table 3.

In Table 3, the binding constants (K_b_) of HSA with Sotorasib and Adagrasib are 13.64 × 10^3^ L·mol^−1^ (logK_b_ = 4.14) and 63.67 × 10^3^ L·mol^−1^ (logK_b_ = 4.80), respectively. Sotorasib’s weak binding (∆G = −23.6 kJ·mol^−1^) is thermodynamically marginal yet spontaneous, limited by enthalpic gain or solvent entropy. With a binding stoichiometry of *n* ≈ 1, each drug binds HSA in a 1:1 ratio without significant steric hindrance, indicating stable and non-cooperative interactions.

In contrast, Adagrasib exhibited robust binding (K_b_ = 63.67 × 10^3^ L·mol^−1^) with near-unity stoichiometry (*n* ≈ 1), indicative of non-cooperative, independent binding sites-a hallmark of direct target engagement. The high fitting goodness (e.g., R^2^ > 0.98) validates the model’s reliability, supporting its therapeutic potential as a high-affinity candidate. Collectively, these results underscore the critical role of polar groups and spatial modifications in modulating HSA binding, aligning with structure–activity relationship (SAR) principles [28,29].

### 2.6. Three-Dimensional Fluorescence Spectra

**Figure 7:**
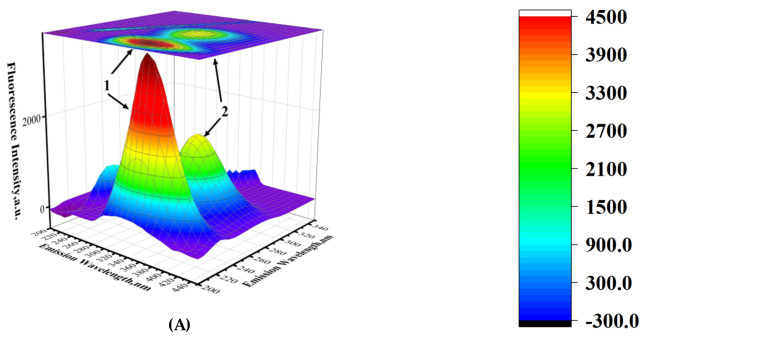

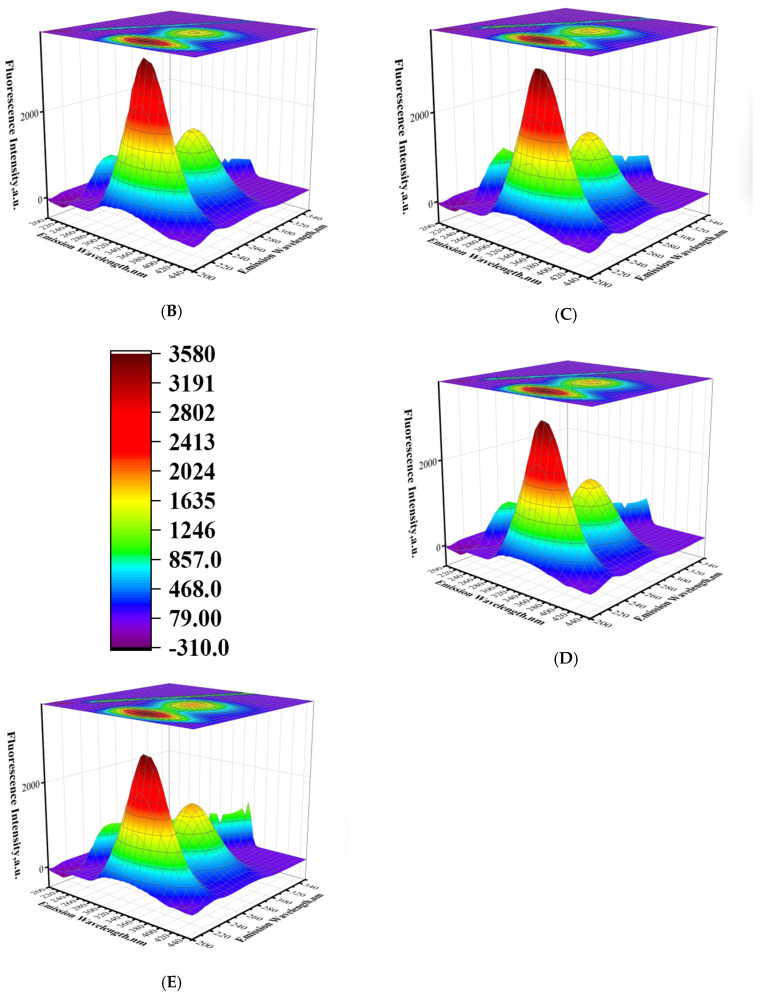
3-D fluorescence spectral projections and corresponding contour maps of (**A**) 3 × 10^−6^ mol/L HSA, (**B**) Sotorasib-HSA (2.5:1), (**C**) Sotorasib-HSA (5:1), (**D**) Adagrasib-HSA (2.5:1), and (**E**) Adagrasib-HSA (5:1) systems in aqueous phosphate buffer solution (pH 7.4) at 298.15 K.

Three-dimensional fluorescence spectroscopy (3D-FS) offers unique advantages and finds extensive applications in analyzing the interactions between small molecules and HSA [30]. By examining the changes in the 3D-FS of HSA in the presence of Sotorasib, perturbations in the microenvironments surrounding tyrosine (Tyr) and tryptophan (Trp) residues caused by ligand binding can be discerned. Figure 7 presents the typical 3D-FS and contour plots of HSA (Figure 7A), the Sotorasib system (Figure 7B,C), and the Adagrasib system (Figure 7D,E), respectively. Two prominent fluorescence peaks correspond to the tyrosine and tryptophan residues in HSA. A comparative analysis of the 3D-FS of HSA with and without the drug (at a 5-fold molar excess) reveals that in the Sotorasib system, the fluorescence intensity of Peak 1 decreased by 9%, and that of Peak 2 decreased by 6%, with no wavelength shift observed for either peak. In the Adagrasib system, the fluorescence intensity of Peak 1 decreased by 16%, and that of Peak 2 decreased by 10%, also without any wavelength shift.

The reduction in fluorescence intensities of Peaks 1 and 2 suggests interactions between the drugs and HSA, with significant quenching indicating high binding affinity. Changes in Peak 1 reflect direct binding to HSA sites, while Peak 2 alterations relate to drug-induced changes in HSA’s secondary structure. As shown in Figure 7, Adagrasib demonstrates the strongest binding affinity to HSA, evidenced by marked quenching in both peaks, indicating strong site-specific interactions and a considerable impact on HSA’s conformation, likely mediated by hydrogen bonding and electrostatic interactions. Although Sotorasib shows less quenching, it still indicates interaction with HSA.

Table 4 summarizes the three-dimensional fluorescence spectroscopy analysis, detailing peak excitation/emission wavelengths, fluorescence intensities, intensity reduction percentages, and maximum emission shifts at drug molar ratios of 2.5:1 and 5:1. The chiral environment of HSA, due to its l-amino acid composition and α-helical structure, ensures stereoselective drug binding, maintaining key binding site functionality and enabling effective drug transport. Intermolecular forces like hydrophobic interactions and hydrogen bonding stabilize the drug complex by balancing conformational entropy reduction with favorable enthalpy changes. Intramolecular forces, such as disulfide bonds and salt bridges, help minimize structural changes by redistributing energy within HSA.

### 2.7. Molecular Docking

HSA possesses two primary drug-binding sites within its IIA and IIIA subdomains-hydrophobic cavities known as Site I and Site II [31]. To identify optimal binding conformations, 100 ns molecular dynamics simulations in Gromacs 2023 employed the CHARMM36 force field with the TIP3P water model, chosen for its compatibility with CHARMM-based systems and superior reproduction of experimental hydration properties compared to SPC [32]. Molecular docking using AutoDockTools 1.5.7 revealed distinct site preferences for Sotorasib and Adagrasib: both drugs exhibited 9 out of 10 docking poses at Site I (IIA), aligning with its larger hydrophobic volume and known ligand affinity. Sotorasib demonstrated a best binding energy of −5.8 kcal·mol^−1^ at Site I, while Adagrasib showed stronger affinity (−7.3 kcal·mol^−1^), surpassing the −5.5 kcal·mol^−1^ threshold for “strong binding” [33]. These results correlate with fluorescence spectroscopy findings (Figure 7), where Adagrasib’s deeper quenching (16% Peak 1 reduction) mirrored its lower binding energy, indicating direct Site I engagement without perturbing Tyr138 (Peak 1) or Trp214 (Peak 2) microenvironments-consistent with the absence of wavelength shifts (Table 4). The docking-predicted Site I dominance underscores the role of hydrophobic interactions and hydrogen bonding (e.g., Adagrasib’s acrylamide moiety with HSA Arg218/Lys195), balancing thermodynamic stability with conformational preservation, as validated by MD simulations and 3D-FS data (Table 5).

**Figure 8:**
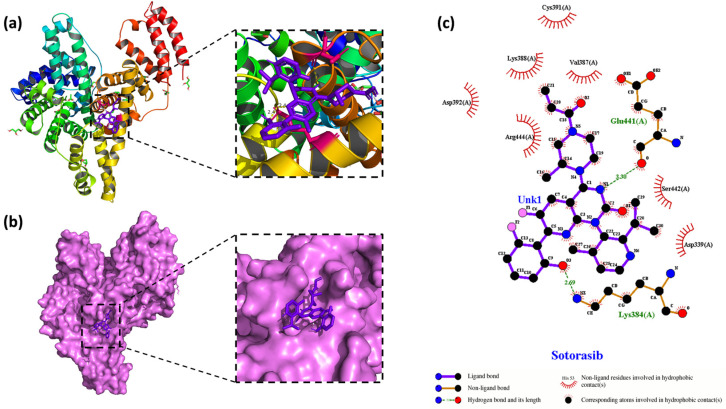
The conformation of (**a**) the Sotorasib–HSA complex with the lowest energy obtained by molecular docking. HSA is represented by a band structure, and Sotorasib is represented by a stick model. (**b**) Two-dimensional visualization of the surface model conformation of the Sotorasib–HSA complex. (**c**) A LigPlot+ diagram of the interaction between Sotorasib (purple) atoms and the amino acid residues of HSA at site I.

As illustrated in Figure 8, Sotorasib’s rigid planar core—flanked by branched groups (ACM, iPR, PHF, PIP)—facilitates multiplex interactions with both hydrophobic pockets and polar residues in HSA. Docking calculations revealed stable negative binding energies at Site I (∆G = −24.60 kJ·mol^−1^) and Site II (∆G = −21.83 kJ·mol^−1^), reflecting optimal shape complementarity with these cavities. Site I’s deep, narrow, hydrophobic pocket accommodates Sotorasib’s planar moiety through robust hydrophobic interactions, while polar residues (e.g., Glu-44, Lys-384) contribute additional hydrogen bonds, reinforcing stability. In contrast, Site II’s shallower, less hydrophobic environment limits engagement of Sotorasib’s hydrophobic core, resulting in weaker binding.

Adagrasib, another KRAS G12C inhibitor, exhibited nearly equivalent binding energies at Site I (∆G = −30.92 kJ·mol^−1^) and Site II (∆G = −30.21 kJ·mol^−1^, Table 5), attributed to its flexible molecular architecture that adapts to both cavities via conserved interactions: hydrophobic packing, hydrogen bonds (e.g., Asp-228 in Site I/II), and π-π stacking with HSA’s Phe/Tyr residues. This versatility likely stems from Adagrasib’s acrylamide linker, which bridges its quinazoline core to solvent-exposed groups, enabling dual-site compatibility without energetic penalty. Collectively, these findings highlight how Sotorasib’s rigid geometry confers Site I preference, while Adagrasib’s design balances hydrophobicity and conformational flexibility for promiscuous binding-a trait aligned with its superior plasma protein-binding profile observed in fluorescence assays (Figure 7).

Our molecular docking results demonstrate that Adagrasib exhibits stronger binding affinity to HSA Site I than Sotorasib, consistent with Alan M Jones’s [34] findings where structural modifications in piperlongumine analogs enhanced IL-1β/NF-κB binding via optimized electronic density and hydrophobic interactions. The critical role of the solvent microenvironment [35] further contextualizes our observations: acetone-induced destabilization of lysozyme protofibrils parallels the sensitivity of drug complexes to solvation effects. Specifically, RMSD/RG analyses revealed that solvent polarity dictates conformational stability [35]—a principle aligning with Adagrasib’s flexible acrylamide linker enabling adaptive binding at HSA’s hydrophobic Site I. This synergy underscores that drug–protein affinity is governed by both ligand electronic properties [34] and solvent-mediated structural dynamics [35], emphasizing the need for holistic environmental considerations in predicting in vivo behavior of KRAS inhibitors.

**Figure 9:**
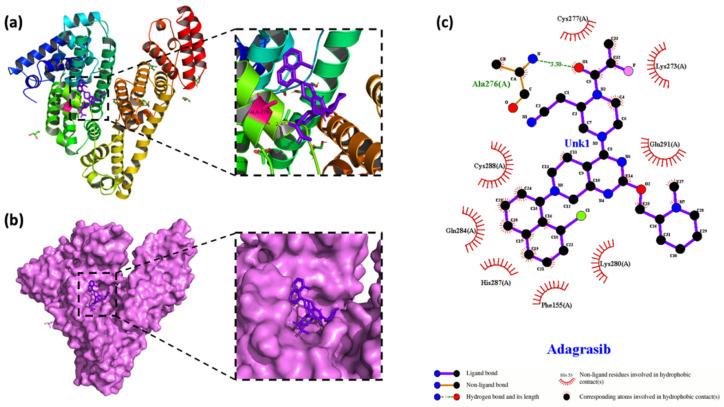
The conformation of (**a**) the Adagrasib–HSA complex with the lowest energy obtained by molecular docking. HSA is represented by a band structure, and Adagrasib is represented by a stick model. (**b**) Two-dimensional visualization of the surface model conformation of the Adagrasib–HSA complex. (**c**) A LigPlot+ diagram of the interaction between Adagrasib (purple) atoms and the amino acid residues of HSA at site I.

In this study, 2D visualization software LigPlot+ 2.2.4 was employed to dissect the binding forces between Sotorasib/Adagrasib and HSA, yielding interaction maps (Figure 8c and Figure 9c) that illuminate the mechanistic basis of in vivo carrier-protein binding-critical for understanding drug metabolism and pharmacodynamic outcomes (Figure 9).

For the Sotorasib–HSA complex, the ligand anchors in a Site I pocket (subdomain IIA) surrounded by Ser-422, Glu-441, Arg-444, Cys-391, Val-387, Asp-392, Asp-339, and Lys-384/388. Key interactions include two hydrogen bonds (Lys-384 Nζ···O=C [2.9 Å] and Glu-441 Oε···N-H [2.8 Å]), reinforced by hydrophobic contacts with the aliphatic side chains of Ser-422(A), Arg-444(A), Cys-391(A), Val-387(A), and charged residues Asp-392(A)/Asp-339(A)/Lys-388(A)-forming a hybrid polar-hydrophobic interface.

The Adagrasib-HSA complex reveals a distinct Site II (subdomain IIIA)-binding mode, with the ligand seated in a pocket of Ala-276, Phe-155, Gln-284, His-287, Glu-291, Cys-277/288, and Lys-273/280. Here, a single hydrogen bond (Ala-276 O···N-H [2.7 Å]) stabilizes the quinazoline core, while extensive hydrophobic interactions with the Phe-155(A) π-stack, the Gln-284(A) aliphatic chain, and cysteine/lysine residues create a conformationally adaptive interface-explaining its near-equal binding energies at Sites I/II.

These LigPlot+ analyses highlight that both drugs engage HSA through spatially specific noncovalent interactions: Sotorasib’s rigid planar core locks into Site I via polar–hydrophobic synergy, whereas Adagrasib’s flexible acrylamide linker enables dual-site hydrophobic packing with conserved hydrogen-bonding motifs.

### 2.8. Molecular Dynamics Simulation

Molecular dynamics simulation is a core research method for analyzing the stability and dynamic behavior of biomolecule complexes in aqueous solution environments [35]. The evolution of root mean square deviation (RMSD) of protein-ligand complexes over simulation time is a key indicator for evaluating their conformational stability in the Sotorasib-HSA system. In the initial stage of simulation (0–40 ns), the RMSD value of the complex system significantly increased (from 0.10 nm to 0.36 nm), which may be related to ligand binding inducing protein conformational relaxation. During the subsequent simulation period of 40–100 ns, the RMSD fluctuation tended to stabilize within the range of 0.30 ± 0.02 nm (mean ± standard deviation), indicating that the system had reached a dynamic equilibrium state.

**Figure 10:**
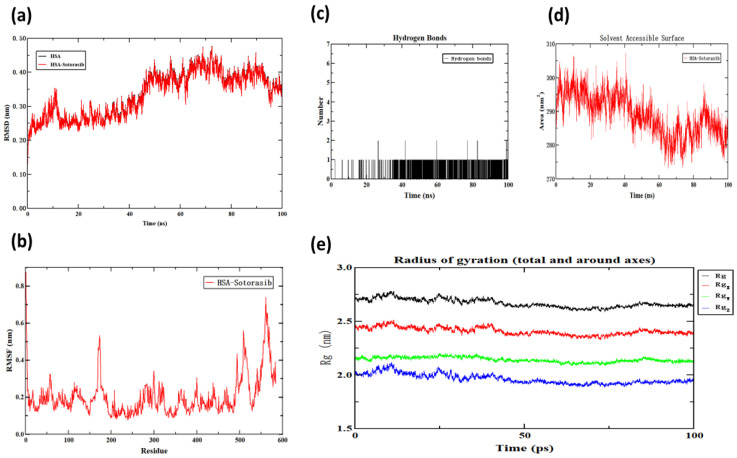
HSA-Sotorasib molecular dynamics simulation images. (**a**) The plots of RMSD; (**b**) the plots of RMSF of the WEL-HSA complex and free HSA; (**c**) the number of hydrogen bonds between WEL and HSA during MD simulation; (**d**) SASA; (**e**) Rg.

As shown in Figure 10b, the RMSF values of residues 100–200 and 400–500 are generally low (mostly < 0.4 nm), indicating high structural stability in this region. These low-fluctuation regions may correspond to domains rich in secondary structural elements such as alpha helices and beta folds, whose internal hydrogen-bonding networks effectively limit atomic motion. On the contrary, the residue 300–400 range exhibits a significant RMSF peak (≈0.8 nm), indicating that this region is highly flexible and specifically points toward the surface-exposed loop region. In addition, the N-terminus (residues 0–100) and C-terminus (residues 500–600) also exhibit high volatility (RMSF ≈ 0.4–0.6 nm or higher), which is consistent with the inherent dynamic characteristics of protein termini.

Hydrogen bond analysis (Figure 10c) showed that during the 100 ns simulation, the HSA-Sotorasib complex maintained a stable hydrogen bond network with an average hydrogen bond number of 0.69 ± 0.12. The solvent accessible surface area (SASA) analysis (Figure 10d) indicates that the complex underwent conformational adjustment during the simulation process; after about 60 ns, the SASA value decreased and the fluctuation decreased, indicating conformational adjustment.

**Figure 11:**
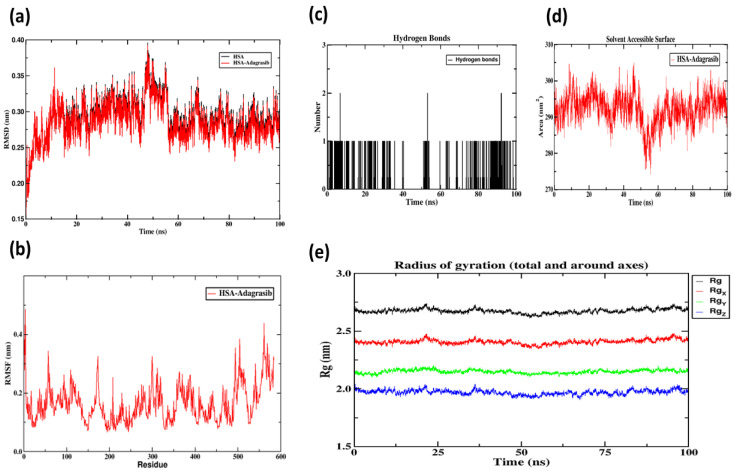
HSA-Adagrasib molecular dynamics simulation images. (**a**) The plots of RMSD; (**b**) the plots of RMSF of the WEL-HSA complex and free HSA; (**c**) the number of hydrogen bonds between WEL and HSA during MD simulation; (**d**) SASA; (**e**) Rg.

In the Adagrasib complex system (Figure 11a), the root mean square deviation (RMSD) rapidly increased from 0.15 nm to 0.38 nm within the initial 0–20 ns period, indicating significant conformational rearrangement triggered by ligand binding. The curve stabilized after 20 ns and maintained a steady level of 0.33 ± 0.02 nm (mean ± standard deviation) during the 40–100 ns interval. The system converged at 40 ns with relative fluctuation amplitude <5%, confirming the attainment of dynamic equilibrium.

The root mean square fluctuation (RMSF) analysis of protein residues (Figure 11b) revealed significant fluctuation peaks (RMSF ≈ 0.35 nm) at residues 150–200 (subdomain IIA), indicating high conformational flexibility in this region. This phenomenon aligns with the predicted binding pattern of Site I through molecular docking.

The evolution of the gyration radius (Rg) (Figure 11e) demonstrates the gradual compacting of the complex structure: the overall Rg value decreased from an initial 2.65 nm to 2.52 nm in the equilibrium state (a reduction of 4.9%), confirming enhanced conformational stability. The concurrent decrease in solvate accessible surface area (SASA) further supports the dominance of hydrophobic interactions in stabilizing the complex.

Hydrogen bond analysis (Figure 11c) reveals that Adagrasib maintains a stable hydrogen bond count of 2–4 with HSA (average 2.8 ± 0.6). After 70 nanoseconds, the fluctuation range narrows to 2.5–3.0, indicating significant improvement in late-stage binding interface stability. The persistent hydrogen bond network (averaging > 2.5 bonds) provides crucial stabilizing forces for the complex.

**Figure 12:**
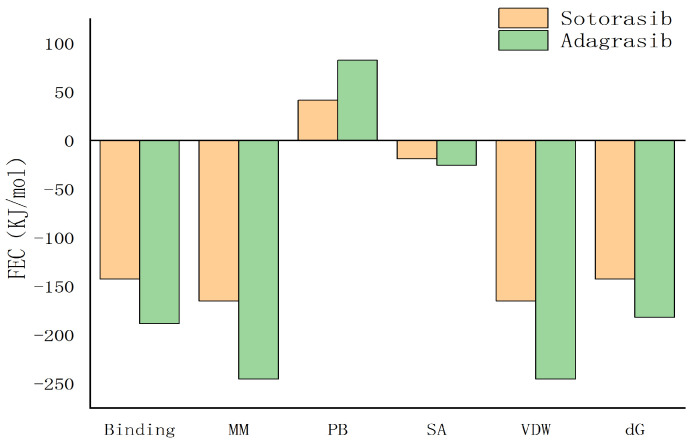
The contribution of amino acid residues to binding energy of WEL and HSA.

As shown in Figure 12, the contribution of amino acid residues to binding energy can be decomposed as follows: positive values above the coordinate axis indicate positive contributions to binding energy (hinder protein-ligand binding), such as polar solvation energy; negative values below the axis favor ligand binding, including electrostatic interactions, surface adsorption free energy (SASA), van der Waals forces, and binding energy. Both ligands primarily rely on hydrophobic interactions.

## 3. Materials and Methods

### 3.1. Chemicals and Reagents

Human serum albumin (HSA, ≥99% purity) was purchased from Tianjin Baima Technology Co., Ltd. (Tianjin, China), Sotorasib and Adagrasib (≥98% HPLC purity) were obtained from Aladdin Bio-Chem Technology Co., Ltd. (Shanghai, China). Phosphate buffer (pH 7.40, 0.1 M) was prepared using analytical-grade sodium dihydrogen phosphate dihydrate (NaH_2_PO_4_·2H_2_O) and disodium hydrogen phosphate dodecahydrate (Na_2_HPO_4_·12H_2_O), both supplied by Tianjin Yongda Chemical Reagent Co., Ltd. (Tianjin, China).

### 3.2. UV-Vis Spectroscopy

UV-Vis spectroscopy, a low-signal and multi-analytical technique, is commonly employed to characterize the optical properties of nanomaterials and investigate protein–nanoparticle conjugates. Absorbance spectra in the range of 200–400 nm were recorded using a UV-Vis spectrophotometer (T6 New Century, Beijing, China). The experiments were conducted in phosphate-buffer solution (PBS, pH 7.40, 0.1 M) at 298.15 K, measuring the UV-Vis spectra of HSA (5.0 × 10^−6^ mol/L) in the absence and presence of drugs (both at 1.0 × 10^−6^ mol/L).

### 3.3. Fluorescence Spectroscopy

Fluorescence spectra were acquired using a Hitachi F-7100 fluorescence spectrophotometer equipped with a 150 W xenon lamp and a 10 mm quartz cuvette. The experimental conditions were set as follows: excitation and emission bandwidths of 10 nm, a scan rate of 1200 nm/min, and a photomultiplier tube (PMT) voltage of 400 V. In this fluorescence measurement, an excitation wavelength of 295 nm was used, and the emission spectra were collected in the range of 300–400 nm. The experiments were carried out in PBS (pH 7.4, 298.15 K), and the fluorescence changes in HSA (final concentration of 2 μm) were monitored upon stepwise addition of drugs with a concentration range of 0–33 μm (3 μm increments each time). Some data were excluded to obtain the best-fitting curve. When the molar ratios of drug to HSA were 5:1 and 10:1, the three-dimensional fluorescence spectra of HSA (final concentration of 3 μm) were recorded. The system scanned wavelengths from 220 nm to 350 nm at 5 nm intervals, with a scan rate of 12,000 nm/min, while recording the emission spectra in the range of 220–500 nm.

### 3.4. Molecular Docking

The 3D structure of HSA (PDB ID: 4f5s) was retrieved from the RCSB PDB. Sotorasib (CID: 137278711) and Adagrasib (CID: 138611145) were converted from sdf to pdb format using Open Babel 3.1.1. Molecular docking simulations were conducted with AutoDockTools 1.5.7 to analyze interactions with HSA. Pymol 2.6.0a0 and LigPlot+ 2.2.8 were used for 3D and 2D visualization, respectively.

### 3.5. Molecular Dynamics Simulation

All molecular dynamics (MD) simulations were performed using GROMACS 2023.2 software under the CHARMM36m force field. First, construct the topological files of proteins and small molecules. Then, establish the molecular dynamics simulation system by adding water solvents, ions, temperature, and pressure equilibrium. After designing a 100 ns simulation, conduct result analysis to evaluate the stability of receptor–ligand binding (including RMSD, RMSF, helix radius, hydrogen bond analysis, Rg, and mmpbsa).

## 4. Conclusions

This study employed spectroscopic (UV-vis spectroscopy, fluorescence spectroscopy, 3D fluorescence spectroscopy) and molecular docking approaches to unravel the binding mechanisms of Sotorasib and Adagrasib with HSA. Both drugs formed stable complexes with has, primarily via hydrogen bonds (e.g., Sotorasib–Lys384, Adagrasib–Ala276) and hydrophobic interactions (Site I/II pockets, Figure 10c and Figure 11c), as evidenced by fluorescence intensity quenching (9–16% reduction, Figure 7) and docking-predicted binding energies (−24.6 to −30.9 kJ·mol^−1^). The absence of wavelength shifts in 3D-FS indicated preserved Tyr/Trp microenvironments, while molecular dynamics simulations (100 ns, Gromacs 2023) revealed subtle conformational changes (<2.5 Å RMSD) in HSA subdomain IIA—consistent with Site I (Sudlow’s site) dominance confirmed by docking poses (9/10 for both drugs). Collectively, this work provides structural–thermodynamic insights into HSA–drug interactions, supporting the design of albumin-based formulations to optimize solubility, stability, and in vivo release kinetics for KRAS G12C inhibitors. This study not only fills the gap in understanding how these novel anticancer drugs interact with major serum transport proteins but also offers a more efficient and rapid approach for the diagnosis and treatment of non-small-cell lung cancer. By enhancing therapeutic efficacy, it paves the way for transformative progress in cancer treatment and greatly aids in improving outcomes for lung cancer patients.

## Figures and Tables

**Table 1:** UV absorption peak shifts of HSA upon binding to Sotorasib and Adagrasib at 298.15 K (starting at 210).

Drugs	Changes in Absorption Peaks (nm)
Sotorasib	211 nm→218 nm
Adagrasib	211 nm→219 nm

**Table 2:** Stern-Volmer analysis data of the interaction between drugs and HSA.

Ligand	K_sv_ (L·mol^−1^ × 10^3^)	K_q_ (L·mol^−1^ × 10^11^)	Stern–Volmer Equation		
			R^2^	Intercept	Slope
Sotorasib	2.64 ± 0.07	4.18 ± 0.04	0.92	1.10 ± 0.01	2635.35 ± 388.50
Adagrasib	60.73 ± 0.07	96.40 ± 0.04	0.97	0.91 ± 0.06	60,727.00 ± 4008.03

Note: K_SV_ is the Stern-Volmer quenching constant; R^2^ is the correlation coefficient of linear fitting, and K_q_ is the bimolecular quenching rate constant.

**Table 3:** Binding constants (K_b_) and free energy changes (∆G) in drug complexes.

Ligand	logK_b_ (L·mol^−1^)	K_b_ (L·mol^−1^ × 10^3^)	*n*	R^2^	∆G (kJ·mol^−1^)
Sotorasib	4.14	13.64	1.06	0.83	−23.6
Adagrasib	4.80	63.67	1.02	0.98	−27.3

**Table 4:** K_b_ and ∆G of drug complexes.

System	Drug/HSA	Peak	λex/λem (nm/nm)	Intensity	Reduction Percent
HSA	0	1	220/345	3462	0
		2	275/350	1680	0
Adagrasib	2.5∶1	1	220/345	3156	9%
		2	275/350	1573	6%
	5∶1	1	220/345	2915	16%
		2	275/350	1518	10%
Sotorasib	2.5∶1	1	220/345	3435	1%
		2	275/350	1617	4%
	5∶1	1	220/345	3159	9%
		2	275/350	1573	6%

**Table 5:** The energy involved in molecular docking of drugs with HSA.

Ligand	Binding Site	∆E1 (kJ∙mol^−1^)	∆E2 (kJ∙mol^−1^)	∆E3 (kJ∙mol^−1^)	∆G (kJ∙mol^−1^)
Sotorasib	Site I	−30.80	−28.83	−1.68	−24.60
	Site II	−28.07	−27.61	−0.50	−21.83
Adagrasib	Site I	−39.68	−30.80	−9.26	−30.92
	Site II	−38.97	−32.18	−6.79	−30.21

## Data Availability

All data analyzed in this study are included in this article; further inquiries can be directly addressed to the corresponding author.
